# A multicenter clinical study on transanal endoscopic sphincter-preserving resection of low rectal tumors

**DOI:** 10.3389/fsurg.2026.1790147

**Published:** 2026-06-12

**Authors:** Xiaoke Hu, Shuaishuai Fan, Jia Xue, Hongrui Li, Guangya Sun, Zeyu Cai, Wang Le, Ruixue Wang, Haiqing Hu

**Affiliations:** 1 The First College of Clinical Medicine, Inner Mongolia Medical University, Hohhot, China; 2 Department of Gastroenterology, Digestive Endoscopy Center, Affiliated Tumor Hospital of Inner Mongolia Medical University (Inner Mongolia Hospital of Peking University Cancer Hospital), Hohhot, China

**Keywords:** clinical outcome, low-grade rectal tumor, multicenter study, sphincter-preserving, transanal endoscopic resection

## Abstract

**Introduction:**

Transanal Endoscopic Sphincter-Preserving Resection (TA-ESPR) is a super-minimally invasive endoscopic technique for digestive tract tumors. This study aimed to evaluate the feasibility, safety, and clinical efficacy of TA-ESPR in patients with low rectal tumors—including elderly individuals with severe comorbidities, postoperative recurrent cases, radiotherapy-resistant residual tumor patients, and those refusing conventional surgery to preserve anal function—and to provide an ultra-minimally invasive treatment option for this patient population.

**Methods:**

A retrospective case series study was conducted. Thirty-seven patients with rectal tumors who underwent TA-ESPR and completed regular follow-up between June 2020 and January 2025 were enrolled from two tertiary medical institutions. All patients declined radical surgery and opted for TA-ESPR after full informed consent; postoperative management was guided by histopathological results.

**Results:**

Among the 37 patients, 73% had low-grade rectal cancer, 16.2% had neuroendocrine tumors, and 10.8% had stromal tumors. The en bloc resection rate and complete resection rate both reached 100%, with no severe adverse events. The mean follow-up duration was 36.86 ± 16.71 months (as of November 1, 2025), and the recurrence rate was 2.7%.

**Conclusion:**

TA-ESPR is a safe and effective treatment for low rectal tumor patients with clinical dilemmas. It effectively preserves anal function, improves postoperative quality of life, and serves as a preferred treatment with important clinical value.

## 1 Introduction

The diagnosis and treatment of rectal tumors may involve imaging evaluation, pathological assessment, endoscopy, surgery, chemoradiotherapy, targeted therapy, and immunotherapy (1). Surgical resection remains the cornerstone of rectal tumor treatment. The lower rectum (defined as the rectal segment ≤5 cm from the anal margin) possesses complex anatomical structures (adjacent to organs such as the anal sphincter, prostate, or vagina). Total mesorectal excision (TME) serves as the gold standard for radical resection of low rectal tumors, requiring complete removal of the rectal mesentery. While achieving favorable oncological outcomes, this procedure may lead to complications such as permanent stoma formation, fecal incontinence, or sexual dysfunction, significantly impacting patients' quality of life (2, 3).

The issue of overtreatment with radical surgery is increasingly evident, particularly for early-stage rectal tumors (confined to the mucosal and submucosal layers) or locally advanced tumors that have undergone neoadjuvant therapy leading to downstaging. The treatment of low rectal tumors often requires a balance between radical resection and functional preservation. Comprehensive consideration should be given to tumor stage, pathological type, the patient's general condition, and the need for function preservation, so as to achieve oncologically radical resection while maximally preserving anal and urogenital function, thereby improving the postoperative quality of life of patients (1).

Local resection has great potential in the organ-preserving strategy for rectal tumors (4). For certain early-stage rectal tumors, local resection has demonstrated favorable therapeutic outcomes. Compared to radical surgical resection, it offers advantages such as reduced trauma, fewer postoperative complications, and higher postoperative quality of life. However, local resection carries a relatively higher risk of residual disease, increasing the likelihood of postoperative recurrence. Adjuvant chemoradiotherapy has been proven to offer local control and survival benefits in rectal tumors, reducing recurrence and metastasis risks while enhancing tumor eradication. Combining local resection with adjuvant chemoradiotherapy represents a viable alternative treatment option for rectal tumors (5, 6).

Endoscopic techniques demonstrate exceptional advantages in the local resection of gastrointestinal lesions. Common endoscopic procedures such as Endoscopic Mucosal Resection (EMR) and Endoscopic Submucosal Dissection (ESD) have become the preferred treatment options for early-stage gastrointestinal tumors. However, both EMR and ESD are limited by the depth of submucosal dissection, making them unsuitable for safely managing severe submucosal fibrosis, muscularis propria adhesions, or invasive lesions. Direct conversion to radical surgery for such conditions may deprive patients of organ-preserving opportunities (7). Based on this clinical dilemma, our center has innovatively proposed and clinically applied TA-ESPR as an important complementary strategy to surgical therapy.It provides a novel ultra-minimally invasive treatment option for patients with low-rectal tumors, including elderly patients with severe comorbidities, those with postoperative recurrence, those with residual local tumors after radiotherapy, and those who refuse conventional surgery due to a strong desire for anal sphincter preservation. However, studies on TA-ESPR remain relatively scarce at present. Further accumulation of high-quality data and long-term oncological outcomes is required to verify its oncological safety. We performed TA-ESPR procedures with caution after thorough informed consent discussions with the aforementioned specific patients with rectal tumors. This article analyzes and reports the clinical data of these patients and the clinical experience obtained from the treatment.

## 2 Patients and methods

### 2.1 Methods

This case series study analyzed 37 patients with low rectal tumors who underwent TA-ESPR treatment and received regular follow-up at the Affiliated Hospital of Inner Mongolia Medical University and the Affiliated Cancer Hospital of Inner Mongolia Medical University between June 2020 and January 2025. These patients were preoperatively diagnosed with low rectal tumors based on digital rectal examination, endoscopy, contrast-enhanced rectal MRI, contrast-enhanced whole-abdominal CT, and transrectal endoscopic ultrasound. The lesions were deemed inoperable via endoscopic mucosal resection (EMR) or endoscopic submucosal dissection (ESD). Patients and their families were fully informed about the TA-ESPR procedure, its potential benefits, risks, and alternative treatments. These patients declined radical surgical resection. Following discussions between the attending physicians and patients, a mutual decision was made to proceed with endoscopic resection. Subsequent treatment plans were determined based on postoperative histopathological evaluation.

### 2.11 Inclusion criteria

1. Tumor invasion confined to the mucosal layer or submucosal layer.
2. Lesion located within 5 cm of the anal margin.
3. Tumor invasion depth reaching the muscularis mucosae or deeper, with strong desire to preserve the anus and refusal of surgical intervention.
4. Informed consent obtained.

#### 2.1.2 Exclusion criteria

1. Failure to obtain patient consent.
2. Patient non-compliance.
3. Bleeding tendency or current anticoagulant use.
4. Severe cardiopulmonary disease precluding endoscopic treatment.
5. Unstable vital signs.

### 2.2 TA-ESPR surgical procedure

**Figure 1:**
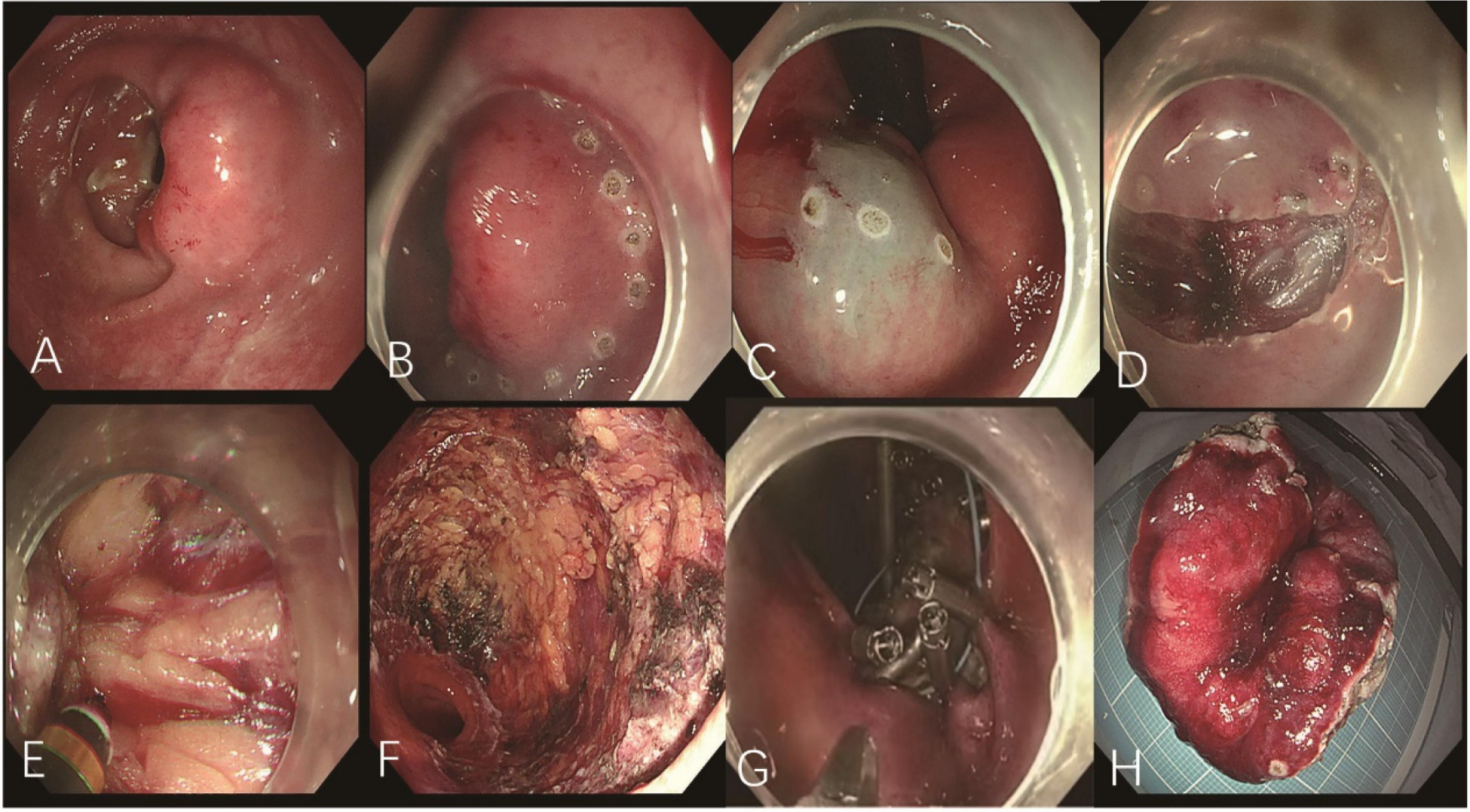
Transanal sphincter-preserving resection. **(A)** Rectal tumor; **(B)** Preoperative marking of lesion; **(C)** Submucosal injection; **(D)** Pre-incision; **(E)** Tumor dissection; **(F)** Wound preparation; **(G)** Suturing; **(H)** Specimen.

The procedure is performed by the same experienced endoscopist with the patient awake:

1. Standard preoperative preparation, including bowel cleansing, is completed while the patient remains fully conscious.
2. The patient assumes a left lateral position (with adjustments as needed during the procedure). A thorough examination is performed by advancing the endoscope through the anus to the ileocecal junction. The scope is then retracted to the tumor site in the rectum for endoscopic visualization of the lesion (Figure 1A).
3. Pre-resection marking: Apply argon plasma coagulation (APC) around the tumor margin, creating a 0.5 cm marked border outside the lesion to guide resection (Figure 1B).
4. Submucosal injection: A mixture of normal saline, 1:10000 epinephrine hydrochloride, and methylene blue was used. Multiple submucosal injections were administered inside and outside the marking dots and repeated as necessary to sufficiently lift the mucosa, resulting in clear tissue layers and easy identification of the lesion (Figure 1C).
5. Pre-incision: Using a gold knife, perform a circumferential pre-incision 0.5 cm outside the marked points through the mucosa and submucosa surrounding the tumor (Endocut mode, effect3, output power 50 W) to precisely expose the tumor (Figure 1D).
6. Reassessment: Based on preoperative endoscopic ultrasound, CT, and MRI findings, combined with intraoperative assessment of tumor size, shape, origin layer, and degree of submucosal fibrosis, reassess whether full-thickness resection is indicated.
7. Tumor Resection: For tumors with significant submucosal fibrosis or those involving the muscularis propria, the tumor should be completely dissected as far away from the tumor mass as possible under good surgical visualization and resected in full thickness. During resection, normal mucosa should be preserved as much as possible, provided that complete tumor resection is ensured, to facilitate suturing of the defect in the intestinal wall (Figure 1E).
8. Specimen Retrieval: Carefully remove the resected tumor specimen in its entirety.
9. Wound Management: Apply electrocoagulation with hemostatic forceps to bleeding sites and exposed small vessels. Confirm the absence of bleeding or oozing to prevent postoperative hemorrhage (Figure 1F).
10. Suturing: Under direct endoscopic visualization, suture the wound using metal clips and nylon sutures. First, insert the nylon suture through the endoscopic channel, open the suture loop to adjust its position, then insert metal clips through the channel. Secure the nylon suture with multiple metal clips along the wound margin, and finally tighten the suture to close the defect (Figure 1G). During suturing, small surgical wounds can be directly closed with complete suturing; for larger wounds, only the defect of the muscularis propria is repaired by suturing.
11. After the tumor specimen has been completely removed, secure it properly on a specimen mounting board and accurately measure the specimen's maximum diameter and relevant dimensions (Figure 1H).
12. During TA-ESPR procedures, ensure a clear field of view at all times and perform the procedure under direct visualization to minimize complications such as tumor residue, bleeding, and damage to adjacent tissues and organs.

### 2.3 Postoperative management and monitoring

Patients should fast for 72 h postoperatively and gradually transition to a liquid diet. Antibiotics should be administered appropriately based on the extent and depth of the surgical resection, and patients should be monitored for complications such as abdominal pain, bleeding, anal pain, infection, and fecal incontinence.

### 2.4 Alternative treatment options

If postoperative pathology indicates high-risk factors for lymph node metastasis (e.g., poorly differentiated tumor, deep submucosal invasion, positive margins, nerve infiltration, or vascular/lymphatic involvement), additional radical surgery is recommended. If the patient still declines radical surgical treatment, adjuvant therapy should be administered based on the pathological findings.

### 2.5 Monitoring strategy

**Figure 2:**
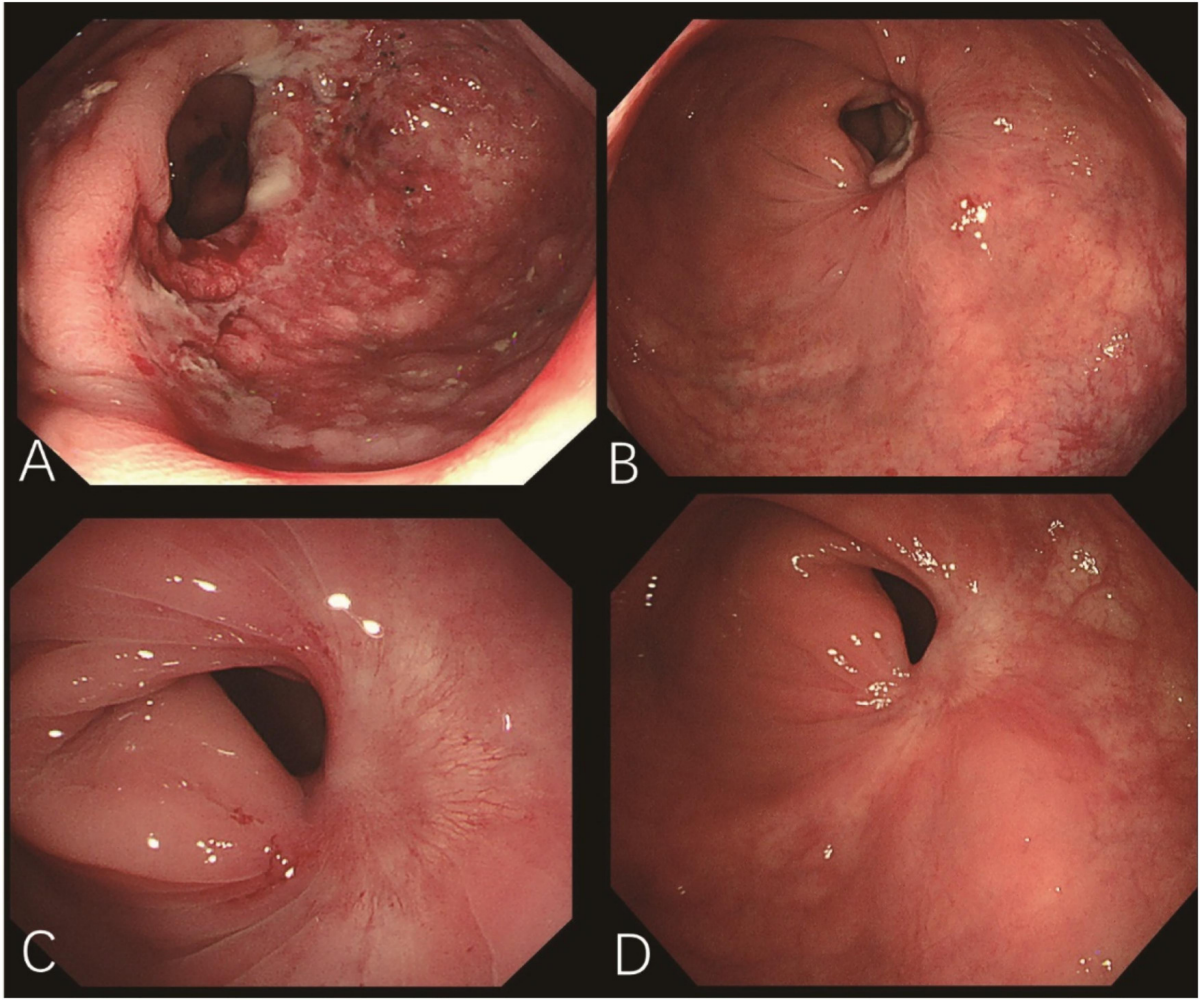
Colonoscopy follow-up. **(A)** 10 days post-surgery; **(B)** 1 month post-surgery; **(C)** 3 months post-surgery; **(D)** 6 months post-surgery.

During the first year post-surgery, patients undergo colonoscopy, contrast-enhanced abdominal CT, chest CT, and tumor marker monitoring at 3, 6, and 12 months to detect recurrence or metastasis. Colonoscopy, as a key postoperative surveillance modality (Figure 2), is prioritized for direct mucosal evaluation. After the first year, monitoring is conducted every 6 months, with immediate intervention as necessary.

### 2.6 Efficacy evaluation

The efficacy of TA-ESPR was assessed based on *en bloc* resection rate, complete resection rate, complication incidence, and postoperative follow-up outcomes (recurrence/metastasis rate).

### 2.7 Statistical methods

Statistical analysis was performed using SPSS 22.0 software. Normally distributed data are expressed as X ± SD, non-normally distributed data as median (IQR), and count data as *n* (%). *P* < 0.05 was considered statistically significant.

## 3 Results

### 3.1 Patient and tumor characteristics, surgical data

A total of 37 patients underwent TA-ESPR. The mean age was 62.3 ± 12.7 years. The median distance from the tumor to the anal margin was 3.0 (2.0–4.0) cm. Preoperative assessment of primary tumor depth of invasion was performed using contrast-enhanced rectal MRI, contrast-enhanced whole-abdominal CT, and transrectal endoscopic ultrasound. En bloc resection and complete resection rates were both 100%. The median tumor diameter was 4.0 (3.5–5.0) cm. For detailed information, please refer to Tables 1 and 2.

**Table 1:** Patient and lesion characteristics.

Patient characteristics(*n* = 37)	Value
Average age	62.3 ± 12.7
Gender	
Male	25
Female	12
TA-ESPR Applicability	
Rectal Cancer	27
Neuroendocrine Tumor	6
Gastrointestinal Stromal Tumors	4

**Table 2:** Tumor Status.

Variable	Value
Median Tumor Diameter	4.0（3.5–5.0）
Median Distance from Tumor to Anal Margin	3.0（2.0–4.0）
Vascular and Nerve Invasion (Yes/No)	12/25
Pathological Type	
Adenocarcinoma	19
Squamous Cell Carcinoma	2
High-Grade Intraepithelial Neoplasia	6
Neuroendocrine Tumor	6
Gastrointestinal Stromal Tumor	4
Depth of Invasion	
Mucosal Layer	4
Submucosal Layer	5
Mucosal Muscular Layer	2
Deep submucosa	6
Lamina propria	15
Intramural fat	4
Internal sphincter layer	1

#### 3.1.1 Rectal cancer

This group comprised 27 patients with rectal cancer. Final histology revealed adenocarcinoma (*n* = 19), squamous cell carcinoma (*n* = 2), and high-grade intraepithelial neoplasia (*n* = 6). Among these, 7 patients had T1N0M0, 17 had T2N0M0, and 3 had T3N0M0. The *en bloc* resection rate and complete resection rate were both 100%.

#### 3.1.2 Neuroendocrine tumors

This group comprised 6 patients with neuroendocrine tumors. Final histology revealed 3 cases as G1 and 3 as G2. Among these, 3 demonstrated submucosal layer invasion, 1 involved the muscularis propria, 1 extended into the muscularis mucosae, and 1 case to the perienteral fat layer. All patients achieved 100% *en bloc* resection and 100% complete resection rates.

#### 3.1.3 Leiomyosarcoma

This group included 4 patients with gastrointestinal stromal tumors (GISTs). Final histology revealed 2 cases as low-risk, 1 as high-risk, and 1 as extremely low-risk. Among these, 3 cases demonstrated infiltration depth to the muscularis propria, while 1 case reached the internal sphincter layer. All patients achieved 100% *en bloc* resection and 100% complete resection rates.

### 3.2 Follow-up treatment

Among the 37 patients, 12 showed postoperative pathological evidence of nerve and vascular infiltration. All patients still declined radical surgical resection, and adjuvant therapy was recommended. All 12 patients received adjuvant therapy postoperatively, while the remaining patients did not receive additional treatment.

### 3.3 Adverse events

No severe adverse events occurred during the operation. Seven patients had minor bleeding (maximum approximately 15 mL in one case), which was controlled by hot biopsy forceps. Nine patients experienced severe anal pain during the procedure and received symptomatic treatment with local lidocaine injection. No other serious complications such as severe abdominal pain, infection, or pneumoperitoneum were observed in any patient. All patients recovered and were discharged after routine conservative medical treatment. For detailed information, please refer to Tables 3.

**Table 3:** Adverse events.

Procedure-related adverse events, n%	Value
Abdominal pain	8
Infection	0
Anal pain	9
Pneumoperitoneum (intraoperative/postoperative)	5/0
Fecal incontinence	0
Bleeding	7

### 3.4 Follow-up results

**Figure 3:**
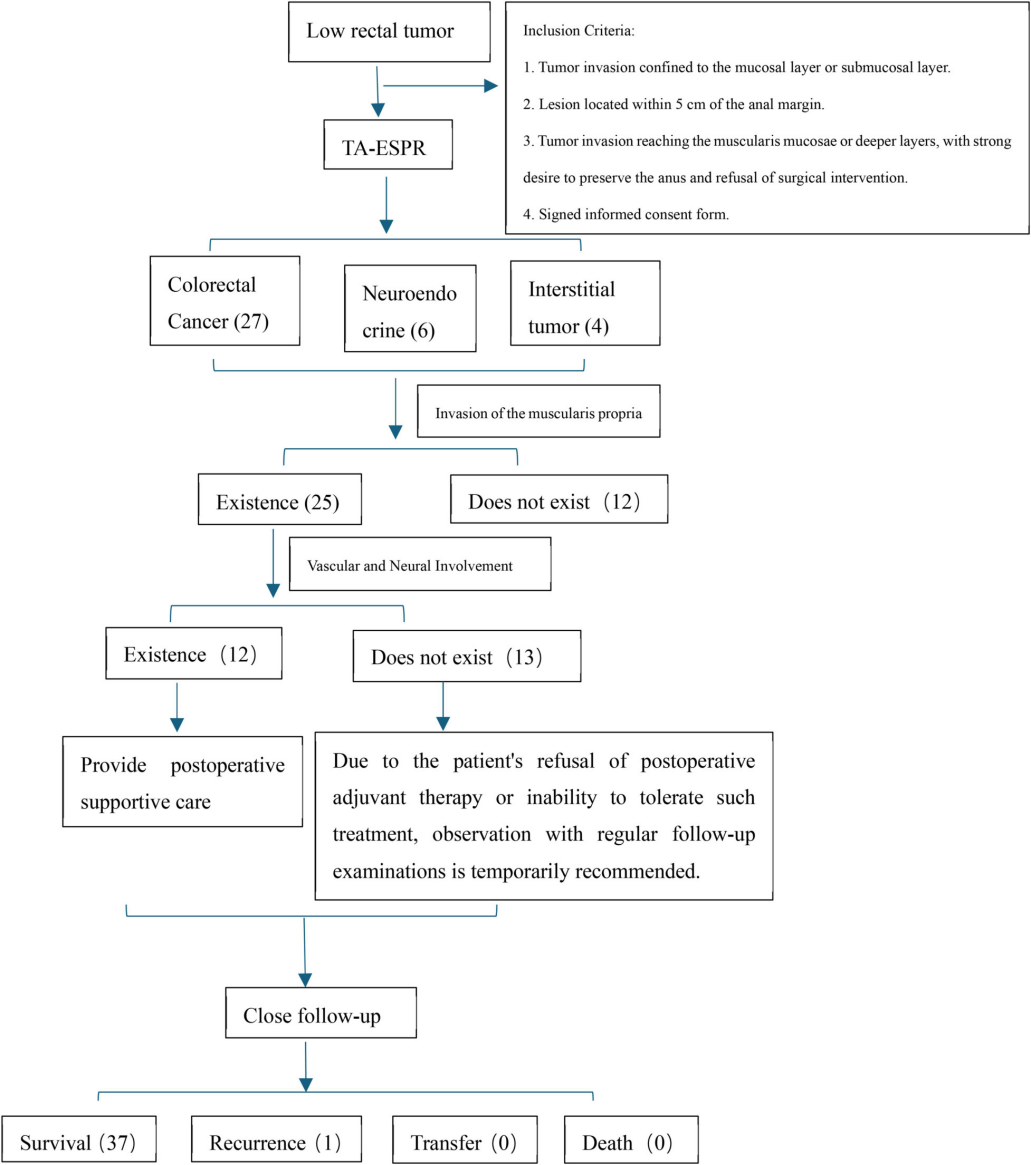
TA-ESPR flowchart.

As of November 1, 2025, patient follow-up data were collected through institutional medical records, survey reports, and telephone follow-ups. The mean follow-up duration was 36.86 ± 16.71 months, with the longest follow-up period reaching 63 months. By the end of this follow-up period, 36 patients showed no recurrence or metastasis, while 1 patient experienced recurrence at 12 months post-surgery due to refusal of adjuvant therapy. For detailed information, please refer to Tables 4. As shown in Figure 3, the above process is illustrated in a flow diagram.

**Table 4:** Baseline information at follow-Up.

Number of valid follow-up cases (*n* = 37)	Value
Number of patients effectively followed up	37
Average follow-up duration	36.86 ± 16.71
Follow-up rate	100%
Long-term outcomes	
Tumor recurrence	1
Tumor metastasis	0
Survival	37
Death	0

## 4 Discussion

Due to the unique anatomical location of low rectal tumors, traditional surgical procedures often require sacrificing anal function, which severely compromises patients' quality of life postoperatively. For elderly patients, those with severe comorbidities, or patients who refuse stoma creation, conventional surgery has clear limitations. This study focused on patients facing such clinical therapeutic dilemmas and performed sphincter-preserving treatment using the TA-ESPR technique. Its core advantages lie in three aspects: minimal invasiveness, sphincter preservation, and safety. First, the procedure is performed via the transanal approach without abdominal incisions, resulting in minimal trauma and low blood loss. It significantly reduces the risk of severe postoperative complications such as infection and intestinal fistula, making it more suitable for elderly patients and those with multiple comorbidities and poor tolerance. Second, the technique prioritizes maximal preservation of anal function. Precise endoscopic local resection of the tumor effectively avoids the need for stoma creation associated with radical surgery. In this study, all patients maintained normal defecation function after surgery, leading to a marked improvement in their postoperative quality of life. Third, the results of this study demonstrate that TA-ESPR achieves a high rate of complete tumor resection, with no severe perioperative complications. This confirms the reliable clinical safety of TA-ESPR in patients with low rectal tumors.

Combined with current advances in clinical research, endoscopic sphincter-preserving procedures have become an important treatment modality for low rectal tumors (8, 9). The TA-ESPR procedure expands the resection range beyond that of conventional endoscopic resection and offers significant clinical advantages for patients with low-rectal tumors whose tumors have invaded the submucosal layer or muscularis propria, who strongly desire sphincter preservation, and who refuse surgical intervention. Among the cases enrolled in this study were 6 cases of rectal adenomas with high-grade intraepithelial neoplasia, 19 cases of rectal adenocarcinoma, 2 cases of rectal squamous cell carcinoma, 6 cases of rectal neuroendocrine tumors, and 4 cases of rectal stromal tumors; During endoscopic evaluation, all of these patients were found to have tumor lesions adherent to or originating from the muscularis propria. Since complete resection was difficult to achieve with conventional ESD, the TA-ESPR procedure was adopted instead, ultimately achieving 100% complete tumor resection. These findings further validate the feasibility and clinical value of the TA-ESPR procedure in this patient population with low-lying rectal tumors.

The patients included in this study were all those for whom conventional surgical procedures were contraindicated or who refused such procedures. They encompassed a variety of cases, including elderly patients with comorbidities, postoperative recurrence, and residual disease following radiotherapy. Due to the complexity of their conditions and low physical tolerance, the selection of treatment regimens must balance efficacy and safety. In this study, the TA-ESPR procedure achieved ideal local tumor control in this patient population, with a low recurrence rate during short-term postoperative follow-up. This suggests that for low-grade rectal tumors without distant metastasis, the TA-ESPR procedure can serve as an effective local treatment modality; Additionally, for patients with high-risk tumor factors such as vascular invasion or muscularis propria invasion, this study recommended postoperative adjuvant therapy. Although some patients did not undergo this treatment due to personal reasons, no significant recurrences were observed during short-term follow-up, providing clinical insights for the development of individualized treatment plans for such high-risk patients in the future.

TA-ESPR for rectal tumors remains in the exploratory phase. The TA-ESPR procedure is technically challenging and requires the surgeon to undergo systematic training and possess extensive experience in endoscopy and rectal surgery, as well as proficiency in lesion resection and wound closure techniques. As resection and closure techniques and equipment continue to advance, the clinical applicability of TA-ESPR is likely to improve. Current applications and research must involve a comprehensive assessment of the patient's condition, with objective disclosure of the potential benefits and risks to both the patient and their family. Endoscopists must aim for complete resection and provide accurate postoperative pathological analysis to guide further treatment. Finally, close follow-up is essential for detecting residual or recurrent tumors and enabling timely intervention.

A major limitation of this study is the relatively short follow-up period, which makes it difficult to draw definitive conclusions regarding the long-term oncological safety of TA-ESPR for the treatment of low-grade rectal tumors. Additionally, among the cases included in this study, 25 patients had high-risk tumor factors such as muscularis propria invasion or deep invasion, and 12 patients had concomitant vascular and nerve invasion. Although adjuvant therapy was recommended for all high-risk patients, only 12 patients received and completed treatment; this situation may have a potential impact on oncological prognosis. Only one case of tumor recurrence was observed during the follow-up period of this study. Short-term follow-up data cannot fully reflect the long-term risk of recurrence; therefore, large-scale, long-term follow-up studies are still needed to further validate the long-term oncological safety of the TA-ESPR procedure.

## 5 Conclusion

TA-ESPR is a safe and feasible endoscopic treatment option for patients with low-rectal tumors who face specific clinical challenges, including the elderly with severe comorbidities, patients with poor tolerance for curative surgery, those with postoperative recurrence, patients with residual local tumors following radiotherapy, or those who strongly desire sphincter preservation. Through standardized procedural steps, this technique enables *en bloc*, full-thickness resection of low rectal tumors under direct endoscopic visualization. Furthermore, the combination of metal clips and nylon sutures for wound closure effectively reduces the risk of postoperative complications such as bleeding and perforation. In clinical practice, TA-ESPR not only maximizes the preservation of anal function and improves patients' quality of life but also offers a novel and effective individualized treatment option for patients with low-rectal tumors who are unsuitable for radical surgery. Due to the unique anatomical location of low-rectal tumors, traditional surgical procedures often require sacrificing anal function, which severely impacts patients' postoperative quality of life. However, due to the limited sample size of this study, the long-term efficacy and safety of TA-ESPR require further validation through multicenter studies with larger sample sizes and longer follow-up periods.

## Data Availability

The raw data supporting the conclusions of this article will be made available by the authors, without undue reservation.
